# Life Insurance

**Published:** 1877-06

**Authors:** 


### *LIFE INSURANCE.*

The agents of some of the Life Insurance Companies have expressed dissatisfaction with the article on "Life Insurance" published in the March number of the JOURNAL. We have no desire or intention to excite unjust prejudice against these companies. We believe that Life Insurance Companies *could be* conducted so as to be a blessing to those for whom they are *ostensibly* founded; but when a majority of all such companies doing business in this State seem to be managed solely in the interests of their own officials; when premiums on policies are saved up, often at the cost of great self-denial, and actual deprivation of many things essential to comfort, to be appropriated in the guise of salaries by a pack of sinecures; when the results of legal investigations have shown the existence of a state of affairs—in many companies formerly the most popular—calculated to make us distrustful of men of the highest pretentions to honor and honesty, we have no intention of becoming accessory to their crimes by recommending "life insurance" in its present unsettled condition, or by publishing their "whitewashed" reports. The "Insurance Department" in this JOURNAL will therefore be discontinued until the moribund portion of the life insurance system shall slough off. Then, if there is any company left that is worthy of confidence, we shall make it known to our readers.

